# ‘Queer in Microbiology’: a Microbiology Society members’ endeavour for creating a safe and inclusive environment for LGBTQIA+ microbiologists

**DOI:** 10.1099/mic.0.001468

**Published:** 2024-06-11

**Authors:** Bruno Francesco Rodrigues de Oliveira, I’ah Donovan-Banfield, Rebekah Penrice-Randal, Rowan Casey, Daniel Gonçalves-Carneiro

**Affiliations:** 1 Department of Microbiology and Parasitology, Biomedical Institute, Fluminense Federal University, Niterói, RJ, Brazil; 2 Institute of Infection, Veterinary and Ecological Sciences, University of Liverpool, Liverpool, UK; 3 NIHR Health Protection Unit in Emerging and Zoonotic Infections, Liverpool, UK; 4 TopMD Precision Medicine Ltd, Southampton, UK; 5 School of Medicine, Cardiff University, Cardiff, UK; 6 Department of Infectious Disease, Faculty of Medicine, Imperial College London, London, UK

**Keywords:** LGBTQIA, Microbiology Society, diversity, inclusion

## Abstract

The past decade has seen growing awareness of the challenges faced by LGBTQIA+ scientists, including discrimination in the workplace and the lack of representation. Initiatives such as 500 Queer Scientists, Pride in STEM and the Microbiology Society’s LGBTQIA+ events have been instrumental in promoting inclusivity in science, technology, engineering, mathematics and medicine (STEMM). The Microbiology Society and its members have played a pivotal role in these efforts and summarized here are their initiatives towards safer and more inclusive scientific and research environments. Starting with a series of interviews and blog posts about the experiences of LGBTQIA+ microbiologists in research, the Society has promoted the organization of networking and social events and developed guidelines for creating more inclusive scientific conferences. These initiatives have not only improved the representation and visibility of LGBTQIA+ individuals in microbiology, but have also served as a blueprint for similar efforts in other scientific areas. Nevertheless, despite improvements in some areas, full inclusion of LGBTQIA+ scientists is still hindered by societal and institutional policies around the world. Here, we propose novel measures to support and empower LGBTQIA+ microbiological communities within learned societies.

## Introduction

For the last decade, society has witnessed a broader discussion of the underrepresentation of women, disabled people, and racial, ethnic, sexual and gender minorities in science, technology, engineering, mathematics and medicine (STEMM), which instigated initiatives to tackle issues faced by these groups and promote diversity in scientific research [1,4]. More recently, the LGBTQIA+ community in STEMM has received increasing attention, after data from different surveys showcased that members of the community feel isolated, invisible, unsafe, constantly disrespected and often marginalized in their workplaces [5,7]. In addition, LGBTQIA+ scientists in training have a higher tendency to stop pursuing a career in academia and industry to escape discrimination and harassment [6,8]. This greatly reduces the odds of researchers succeeding and, consequently, hinders the production of high-quality research by a more diverse and globally competitive workforce [9].

Fortunately, some aspects of the wide variety of research ecosystems are gradually changing to raise awareness, bringing more representation to science and championing LGBTQIA+ people working in STEMM. A major achievement is the creation of community-focused networks, organized by LGBTQIA+ scientists primarily in academia during the 2010s [10], notably 500 Queer Scientists (500QS) [11], Pride in STEM [12] and Queer STEM [13]. In parallel, many studies about research culture and inclusiveness were conducted [14,15], launching several initiatives within major scientific areas. These efforts have created spaces in which the LGBTQIA+ scientific community can share their main struggles experienced in the work environment and have opened avenues for the implementation of LGBTQIA+-inclusive policies [16,20]. This sparse but valuable series of ‘first steps’ actions have highlighted the need for official data collection and reporting systems for the LGBTQIA+ community [21] and, most importantly, the delineation of a group of interventions at all levels to increase their visibility in STEMM fields [22]. In light of the changes in research environments, members of learned societies, including the Microbiology Society, have concentrated significant efforts to support the LGBTQIA+ community. While longer and deeper sustained support is required, we believe that our preliminary actions, highlighted in the next section, are an example of how the community can lead to more inclusive environments within learned societies.

## ‘Queer in microbiology’: origins and major actions

With the goal of incorporating Equity, Diversity and Inclusion (EDI) policies in its structure, the Microbiology Society has collected data from its members by means of broad surveys, which were initially focused on gender identity in 2013 and were expanded to also incorporate details on ethnicity, disabilities and sexual orientation in 2018. Focusing on the LGBTQIA+ community, the Society launched an interview series taking place yearly on LGBTQIA+ STEMM Day [23,28] and facilitated blog posts on other community-related Awareness Days [29,34]. In these blogs, LGBTQIA+ members shared their personal and professional experiences as part of the community and its influence on their academic paths. With the goal to broaden the range of experiences represented in these blog posts, a wider call for content from the Society to all of its members would increase representation and engagement with a larger group of scientists, also bringing a more intersectional and urgent aspect to other underrepresented and marginalized groups that LGBTQIA+ members may also belong to.

Underlying the actions aimed at the LGBTQIA+ community within the Microbiology Society is a 2020 survey conducted by the Society’s members to obtain a clearer view of the representation, diversity and needs of LGBTQIA+ microbiologists. A total of 64 respondents participated, most of them based in the UK and/or self-identified as gay men. While 42% of the respondents stated they were out to all of their work colleagues, almost one-third disclosed they were only out to some of their close colleagues and friends. This survey also identified the lack of opportunities to meet other LGBTQIA+ microbiologists. Other relevant feedback highlighted the need for professional development within and beyond the LGBTQIA+ microbiology community, broad adoption of non-gendered language in the Society’s meetings, institutional support for greater visibility of LGBTQIA+ microbiologists, normalization of individual pronoun use and the availability of resources for LGBTQIA+ members.

Based on this feedback, a group of members of the Microbiology Society formed the LGBTQIA+ Working Group to address some of these needs identified by its LGBTQIA+ community. The efforts of this initial working group led to the first LGBTQIA+ networking event, which took place during the Society’s Annual Conference in Belfast, UK, in 2022 [35]. Participants of this social/networking event reported positive interactions with other LGBTQIA+ members and an enhanced feeling of belonging and representation. During this event, the participants also discussed which actions the Microbiology Society may take to support its LGBTQIA+ members while highlighting their research and professional achievements within the umbrella of other historically marginalized groups. One issue raised by the attendees was the absence of openly LGBTQIA+ role models in microbial sciences, especially those in advanced career stages and leadership positions. Finally, the majority of the attendees confirmed their interest in receiving information on future LGBTQIA+ events organized by the Society, which strongly motivated our working group to plan novel actions in that direction.

With the enthusiastic reaction of the participants in the first event, our working group set up a virtual event for the LGBTQIA+ STEMM Day 2022, ‘Queer in Microbiology: A Conversation’ [36]. The event platformed a discussion with a diverse panel of LGBTQIA+ scientists at different career stages, working in academia and industry. Holding an online discussion enabled the participation of many more international researchers, which broadened the diversity of views of the LGBTQIA+ community in microbiology. The members of the discussion panel shared their experiences about feeling comfortable and safe at work, challenges faced throughout their career as openly LGBTQIA+ microbiologists and the benefits of finding community in the workspace. New ideas for improving the current working conditions were also proposed, specifically in terms of training opportunities, mentoring schemes and professional development. The 2h event had a total of 87 attendees, most of whom were not members of the Microbiology Society at the time of the event, and were mostly based outside of the UK, which was a rewarding outcome since it demonstrated that the pro-inclusion actions of the Microbiology Society and its members have a worldwide impact, even outside the Society. Attendees’ feedback demonstrated that such networking opportunities – at the workplace or during scientific meetings – greatly impact the LGBTQIA+ community’s sense of belonging. Moreover, this event also highlighted the need for a wider representation of speakers in terms of ethnic and geographical diversity, neurodivergence, people with disabilities and other minoritized groups.

In particular, this event brought the Society’s member-led LGBTQIA+ Working Group efforts to the attention of other community-organized groups, working towards better inclusion and representation. Such exposure led to opportunities to enrol in international initiatives for systematically promoting and connecting LGBTQIA+ microbiologists. The first of these initiatives, led by an international group of LGBTQIA+ scientists, was published as a perspective piece detailing a comprehensive set of guidelines to organize safer and more inclusive scientific conferences for LGBTQIA+ scientists [37]. This effort involved a cross-institutional collaboration between LGBTQIA+ microbiologists and led to a compendium of actions that organizers can readily incorporate to turn scientific meetings into safer, fairer and more welcoming experiences for queer- and trans-people. Actions such as the generation of spaces for underrepresented groups within the LGBTQIA+ community and the need to adopt an intersectional view were also addressed since these are pivotal for a fundamental restructuring of scientific conferences [37].

The second initiative was the creation of the Pride in Microbiology Network [38], launched in June 2023. This network was created in response to an editorial that highlighted the shared struggle of historically marginalized groups in microbiology [39]. The Pride in Microbiology Network aims to add an LGBTQIA+ perspective to this work by providing a networking platform for LGBTQIA+ microbiologists to promote their research, collaborate, share their ideas on fighting prejudice and disparity in academia and industry, and propose novel policies addressing issues the LGBTQIA+ community faces in the workplace. The mobilization of this international group of scientists is evidence that the pioneering efforts conducted by the Microbiology Society LGBTQIA+ Working Group have positively impacted the global LGBTQIA+ microbiology community, highlighting its strength in increasing visibility and unity.

Our most recent initiative was holding the second LGBTQIA+ networking event during the Society’s Annual Conference in Birmingham, UK [40]. All 47 attendees had the opportunity to interact and discuss their experiences as part of the community and their research interests, opening avenues for scientific collaboration. Whilst overall a positive experience, attendees’ feedback called for more diffusion, wider advertisement of networking opportunities and the need to reach early career researchers, such as undergraduate students. Participants also highlighted the importance of sharing experiences and career prospects from more senior scientists, showcasing the work of LGBTQIA+ microbiologists. These suggestions were all considered as we organized and held the third LGBTQIA+ networking event at the Microbiology Society’s Annual Conference in Edinburgh, UK, in the second week of April 2024. Such feedback is encouraging but is also indicative of a wider demand for more space and greater reach of these networking events.

## Moving forward

Whilst progress has been made towards inclusion, representation and safer workspace conditions in some countries, other parts of the globe still condemn and punish the existence of many members of the LGBTQIA+ community. Additionally, new challenges have arisen for LGBTQIA+ scientists to face, even in countries often labelled as progressive societies. Laws targeting the LGBTQIA+ community have been approved [41], rising rates of hate crimes [42], anti-transgender rhetoric in the media [43] and the restriction to healthcare access pose risks to LGBTQIA+ microbiologists worldwide.

**Fig. 1.**
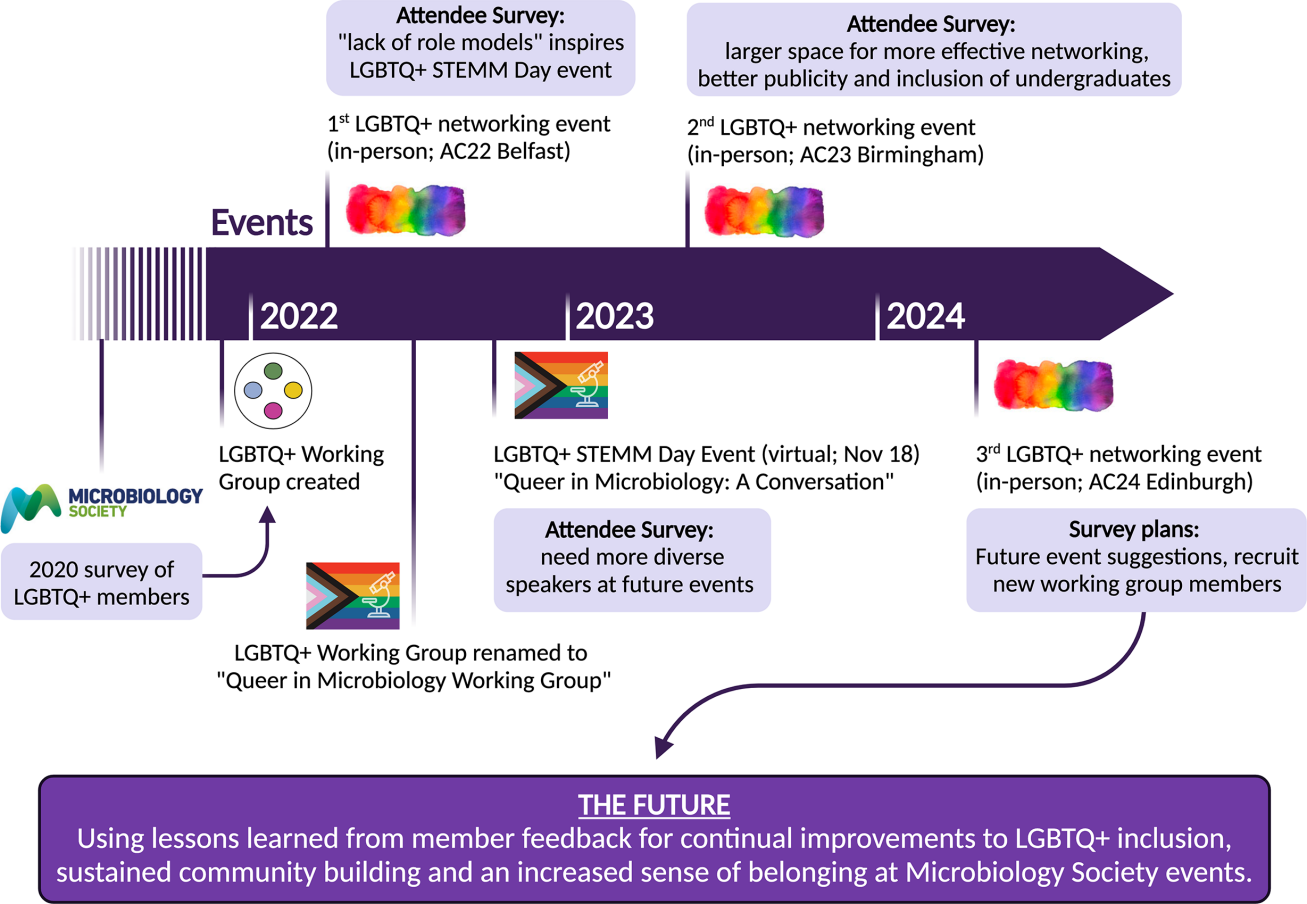
Timeline of the main initiatives organized and under organization by the ‘Queer in Microbiology’ Working Group at the Microbiology Society since 2022. AC: Annual Conference.

International learned societies have an obligation to support all of their LGBTQIA+ members worldwide, to guarantee scientific excellence and the exchange of scientific knowledge. Outreach projects for supporting and connecting LGBTQIA+ researchers in STEMM fields have been gaining momentum, in line with a push to increase diversity in science. Although several initiatives have preceded our efforts, the ‘Queer in Microbiology’ Working Group at the Microbiology Society has been the first to systematically promote activities for LGBTQIA+ microbiologists (Fig. 1). In less than 2 years, our actions have already served as a blueprint for several wider projects for the community, such as the Pride in Microbiology Network [38]. This would not have been achieved without the Society’s EDI policies, the creation of a panel of microbiologists from historically marginalized communities (including LGBTQIA+ members) to consult on EDI topics, and its members’ willingness to make actual change [44,46]. Whilst this recognition has been positive, formalizing the Society’s EDI advisory group (known as the ‘Members’ Panel’) with voting rights within the governance structure would facilitate long-lasting structural improvements to foster a more inclusive society. The establishment of this foundation will stimulate further member-led actions such as creating additional virtual content for the LGBTQIA+ microbiological community. This could include upcoming local and international networking events and initiatives, spotlight features of queer- and trans-microbiologists in different career stages, sign-posting resources for other community-led organizations and additional educational resources. However, such action will be best accomplished by expanding this working group to other members, working closely with the Society Members’ Panel, and closer collaboration with other organized groups globally. Despite increased support for member-led initiatives, we acknowledge that broader structural changes in learned societies are required to directly translate the needs of all its members.

We are seizing this moment to honour LGBTQIA+ microbiologists globally, and reiterate our relentless commitment to ensuring future scientists have abundant access to role models from this community to connect with and draw inspiration from.

## Abbreviations

EDI: equity, diversity and inclusion
LGBTQIA+: lesbian, gay, bisexual, transgender, queer, intersex, asexual
STEMM: science, technology, engineering, mathematics and medicine
UK: United Kingdom
